# Risk factors for foot ulceration in adults with end-stage renal disease on dialysis: a prospective observational cohort study

**DOI:** 10.1186/s12882-019-1594-5

**Published:** 2019-11-21

**Authors:** Michelle R. Kaminski, Katrina A. Lambert, Anita Raspovic, Lawrence P. McMahon, Bircan Erbas, Peter F. Mount, Peter G. Kerr, Karl B. Landorf

**Affiliations:** 1 Discipline of Podiatry, School of Allied Health, Health Services and Sport, La Trobe University, Melbourne, Victoria 3086 Australia; 2 Department of Public Health, School of Psychology and Public Health, La Trobe University, Victoria, 3086 Australia; 3 Departments of Renal Medicine & Obstetric Medicine, Eastern Health Clinical School, Monash University, Melbourne, Victoria 3128 Australia; 4 Department of Nephrology, Austin Health, University of Melbourne, Heidelberg, Victoria 3084 Australia; 5 Department of Nephrology, Monash Health, Melbourne, Victoria 3168 Australia; 6 La Trobe Sport and Exercise Medicine Research Centre, School of Allied Health, Health Services and Sport, La Trobe University, Victoria, 3086 Australia

**Keywords:** Amputation, Chronic kidney failure, Dialysis, Foot ulcer, Risk factors

## Abstract

**Background:**

Dialysis patients experience high rates of foot ulceration. Although risk factors for ulceration have been extensively studied in patients with diabetes, there is limited high-quality, longitudinal evidence in the dialysis population. Therefore, this study investigated risk factors for foot ulceration in a stable dialysis cohort.

**Methods:**

We prospectively collected clinical, demographic, health status, and foot examination information on 450 adults with end-stage renal disease from satellite and home-therapy dialysis units in Melbourne, Australia over 12 months. The primary outcome was foot ulceration. Cox proportional hazard modelling and multinomial regression were used to investigate risk factors.

**Results:**

Among 450 dialysis patients (mean age, 67.5 years; 64.7% male; 94% hemodialysis; 50.2% diabetes), new cases of foot ulceration were identified in 81 (18%) participants. Overall, risk factors for foot ulceration were neuropathy (HR 3.02; 95% CI 1.48 to 6.15) and previous ulceration (HR 2.86; CI 1.53 to 5.34). In those *without* history of ulceration, nail pathology (RR 3.85; CI 1.08 to 13.75) and neuropathy (RR 2.66; CI 1.04 to 6.82) were risk factors. In those *with* history of ulceration, neuropathy (RR 11.23; CI 3.16 to 39.87), peripheral arterial disease (RR 7.15; CI 2.24 to 22.82) and cerebrovascular disease (RR 2.08; CI 1.04 to 4.16) were risk factors. There were 12 (2.7%) new amputations, 96 (21.3%) infections, 24 (5.3%) revascularizations, 42 (9.3%) foot-related hospitalizations, and 52 (11.6%) deaths.

**Conclusions:**

Neuropathy and previous ulceration are major risk factors for foot ulceration in dialysis patients. Risk factors differ between those with and without prior ulceration. The risk factors identified will help to reduce the incidence of ulceration and its associated complications.

## Background

Foot ulceration is a worldwide public health concern that causes significant morbidity [1–5]. Its incidence appears to be accelerated by concurrent diabetes and other common illnesses, such as peripheral arterial disease [3, 4, 6]. Ulcers frequently become infected, limit mobility, and may lead to amputation and mortality [3, 4]. However, when modifiable risk factors are identified and managed early, such complications are often preventable [7, 8].

Although risk factors for ulceration have been extensively studied in patients with diabetes [9, 10], there is surprisingly limited high-quality evidence in the dialysis population, despite an estimated 14% prevalence [11]. Both foot salvage and survival rates are poor in these patients; only half survive 12 months after amputation [3, 4, 12]. We previously reported in a systematic review of existing studies that the strongest risk factors for ulceration in dialysis patients include previous ulceration or amputation, peripheral neuropathy, diabetes and macrovascular disease [11]. However, studies in our review did not provide high-level evidence because of small sample sizes, inadequate appraisal of risk factors or comorbidities, and most were cross-sectional or retrospective. This study aimed to address these deficiencies.

## Methods

Detailed methods have been described elsewhere [13, 14]. This study was approved by the relevant institutional ethics committees and all participants gave written informed consent [13].

### Participants

**Fig. 1:**
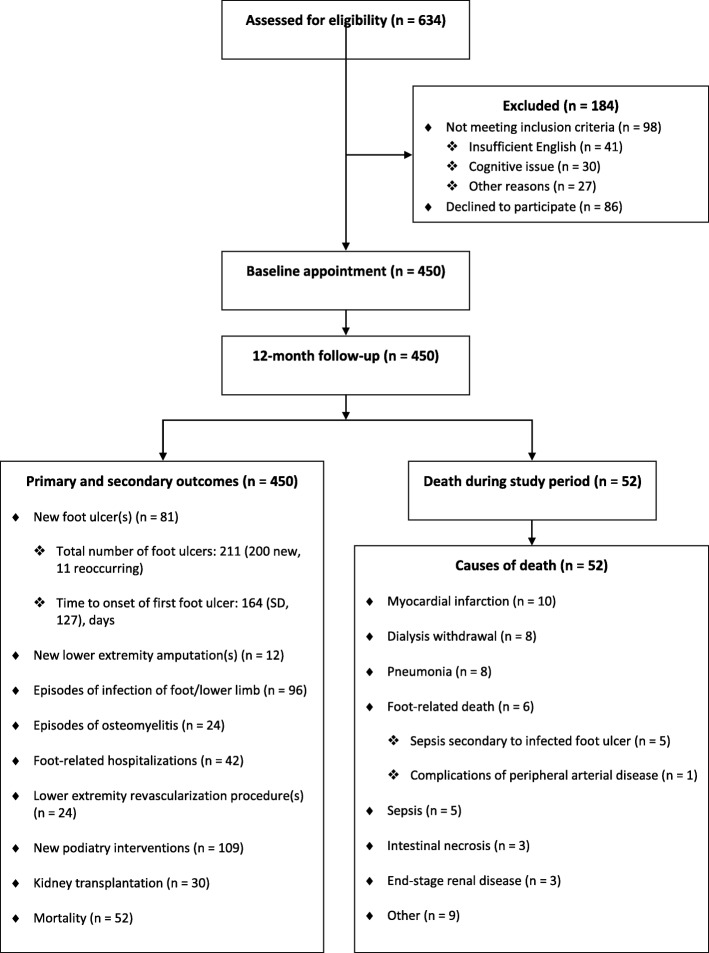
Diagram of participant flow and study outcomes

This multi-center prospective cohort study recruited adults with end-stage renal disease (ESRD) from 13 satellite and home-therapy dialysis units in Melbourne, Australia from January 2014 to December 2015 (Fig. 1 and Table 1). Participants were eligible if they had ESRD and were clinically stable on dialysis (hemodialysis or peritoneal dialysis), aged 18 years or over, and able to provide informed consent (i.e. cognitively aware). Participants were excluded if they had insufficient English language skills to provide informed consent or follow instructions.

**Table 1:** Participant characteristics according to foot ulceration status at follow-up

	Total (*N* = 450)	Foot ulceration		
		Yes (*n* = 81)	No (*n* = 369)	*P*-value*
Time to follow-up, mean (SD), days	366 (8)	366 (7)	366 (9)	0.48
Age, mean (SD), years	68 (13)	69 (10)	67 (14)	0.33
Male sex, n (%)	291 (65)	56 (69)	235 (64)	0.42
BMI, mean (SD), kg/m^2^	28.2 (6.6)	29.9 (7.0)	27.8 (6.4)	0.01*
Current smoking, n (%)	54 (12)	9 (11)	45 (12)	0.93
Cause of ESRD				
Diabetes mellitus, n (%)	180 (40)	53 (65)	127 (34)	< 0.001*
Hypertension, n (%)	28 (6)	4 (5)	24 (7)	0.78
Glomerulonephritis, n (%)	97 (22)	11 (14)	86 (23)	0.08
Polycystic kidney disease, n (%)	22 (5)	4 (5)	18 (5)	> 0.99
Reflux, n (%)	19 (4)	1 (1)	18 (5)	0.24
Renovascular disease, n (%)	10 (2)	3 (4)	7 (2)	0.56
Vasculitis, n (%)	9 (2)	1 (1)	8 (2)	0.92
Unknown, n (%)	15 (3)	0 (0)	15 (4)	0.13
Other, n (%)	70 (16)	4 (5)	66 (18)	0.006*
Dialysis treatment				
Hemodialysis, n (%)	423 (94)	79 (98)	344 (93)	0.85
Peritoneal dialysis				
CAPD, n (%)	9 (2)	1 (1)	8 (2)	0.92
APD, n (%)	18 (4)	1 (1)	17 (5)	> 0.99
Dialysis duration, median (IQR), months	36.9 (16.6 to 70.1)	41.3 (19.7 to 82.1)	36.4 (14.7 to 67.4)	0.28
Diabetes, n (%)	226 (50)	58 (72)	168 (46)	< 0.001*
Type 1, n (%)	13 (6)	6 (7)	7 (2)	0.16
Type 2, n (%)	213 (94)	52 (64)	161 (44)	0.16
Diabetes duration, mean (SD), months	256.3 (152.6)	311.7 (157.2)	237.1 (146.6)	0.002*
Known peripheral neuropathy, n (%)	70 (16)	31 (38)	39 (11)	< 0.001*
Known peripheral arterial disease, n (%)	79 (18)	36 (44)	43 (12)	< 0.001*
Hypertension, n (%)^a^	360 (80)	66 (82)	294 (80)	0.83
Dyslipidemia, n (%)	301 (67)	64 (79)	237 (64)	0.02*
Ischemic heart disease, n (%)	263 (58)	58 (72)	205 (56)	0.01*
Cerebrovascular disease, n (%)	104 (23)	31 (38)	73 (20)	0.001*
CRP, median (IQR), mg/L^b^	7.33 (2.83 to 19.67)	10.33 (4.65 to 24.38)	6.67 (2.67 to 18.75)	0.03
Serum albumin, mean (SD), g/L	33.7 (3.9)	32.8 (4.9)	33.9 (3.7)	0.06
Total calcium, mean (SD), mmol/L	2.20 (0.14)	2.20 (0.15)	2.20 (0.13)	0.86
Serum phosphate, mean (SD), mmol/L	1.55 (0.38)	1.60 (0.44)	1.54 (0.37)	0.27
PTH, median (IQR), pmol/L	29.58 (18.04 to 45.84)	27.53 (21.17 to 45.97)	29.83 (16.93 to 45.65)	0.46
HbA1c, mean (SD), %^b^	6.14 (1.31)	6.65 (1.35)	6.02 (1.28)	< 0.001*
Hemoglobin, median (IQR), g/L	111.3 (102.9 to 117.7)	112.7 (102.8 to 120.2)	111.0 (102.8 to 117.3)	0.30
Previous foot ulceration, n (%)	97 (22)	45 (56)	52 (14)	< 0.001*
Baseline foot ulceration, n (%)	45 (10)	36 (44)	9 (2)	< 0.001*
Baseline amputation, n (%)	46 (10)	30 (37)	16 (4)	< 0.001*

Data are n (%), unless otherwise specified. Percentages may not add up to 100%, as they are rounded to the nearest percent

The complete dataset of participant characteristics can be found in Additional file 1

*SD* Standard deviation, *BMI* Body mass index, *ESRD* End-stage renal disease, *CAPD* Continuous ambulatory peritoneal dialysis, *APD* Automated peritoneal dialysis, *IQR* Interquartile range, *CRP* C-reactive protein, *PTH* Parathyroid hormone, *HbA1c* Glycated hemoglobin

SI conversion factor: To convert CRP to nanomoles per liter, multiply by 9.524. To convert PTH to nanograms per liter, multiply by 9.4. To convert HbA1c to proportion of total hemoglobin, multiply by 0.01

*Significant difference between ‘foot ulceration’ and ‘no foot ulceration’ groups, *p* < 0.05

^a^Requiring medication

^b^Maximum missing data were for glycated hemoglobin (HbA1c) involving 39 participants overall (8.7%). Missing data were for glycated hemoglobin (*n* = 39) and C-reactive protein (*n* = 3)

### Data collection

One examiner (M.R.K.) collected baseline (participant interview, medical record review, health-status and foot examination) and 12-month data (primary and secondary outcomes). Twenty participants were also included in a reliability study to evaluate examiner reliability of the assessment tools [13, 14]. In brief, there was strong intra-examiner reliability for the foot assessments. For continuous data, intra-class correlation coefficients ranged from 0.87 to 0.99. For dichotomous data, all weighted kappa values equalled 1.00 with the absolute percentage agreement ranging from 95 to 100% [14].

### Primary outcome

The primary outcome was the development of a foot ulcer, which was verified by reviewing medical records [13]. Foot ulcers were documented as ‘new’ or ‘reoccurring’, however both were classified and recorded as the primary outcome in this study. *New ulcers* were defined as an ulcer that occurred for the first time during the study period, or, if a participant had an ulcer at baseline, a new ulcer at a different site on the same or contralateral foot during the study period. *Reoccurring ulcers* were defined as a foot ulcer present at baseline that healed and re-ulcerated at the same site during the study.

### Secondary outcomes

Secondary outcomes included: number and time to onset of new foot ulcers and new lower extremity amputations; episodes of foot or lower limb infection, osteomyelitis, and foot-related hospitalizations; lower extremity revascularization procedures; new podiatry interventions; kidney transplantation; and mortality [13]. Time to onset was defined as the ‘number of days between baseline and the development of a new foot ulcer’ [13]. Secondary outcomes were verified by reviewing medical records.

### Sample size

Four hundred and fifty participants were recruited with a pre-specified sample size [13].

### Statistical analysis

Primary and secondary outcome data were calculated and expressed as mean (standard deviation, SD) or median (interquartile range, IQR). Continuous data were checked for normality. To explore between-group differences, independent samples *t*-tests, Mann-Whitney *U* tests and/or Chi-square tests were calculated depending on data type. Unadjusted foot ulcer incidence rates were calculated for number of events per 1000 person-years.

Univariate and multivariate relative risks were estimated by Cox proportional hazard modelling for new cases only (i.e. excluded participants with a baseline ulcer) and were adjusted for peripheral neuropathy, previous foot ulceration and cerebrovascular disease. We performed stratified analyses to assess whether the association between diabetes and risk of ulceration varied. The Nelson-Aalen cumulative hazard estimate and the Kaplan Meier survival estimates were calculated. Univariate modelling included risk factors with *p* < 0.2. We performed a step-wise modelling approach where models were built to exclude *p* > 0.1 and include if *p* < 0.05. The models were checked proportionally with time dependence and Schoenfeld scaled residuals. Goodness of fit was examined with Cox-Snell residuals.

Multinomial logistic regression was used to relate a three-category outcome to screened variables at baseline. Categories included: (i) no development of foot ulceration (no previous or baseline ulceration, and did not develop ulceration) [reference category], (ii) development of foot ulceration (no previous or baseline ulceration, but developed ulceration), and (iii) development of foot ulceration (previous and/or baseline ulceration, and developed ulceration). The multinomial regression model resulted in two sets of odds ratios (OR) for each risk factor and each level of the outcome. Models were adjusted for age, male sex, living alone, podiatry attendance. Risk estimates were presented as relative risk (RR) or hazard ratios (HR) with 95% confidence intervals (CIs). The threshold for statistical significance was set at *p* < 0.05 [13].

We stratified the data by diabetes status to identify possible effect modification. Where indicated, models with interaction terms between diabetes status and other risk factors were considered statistically significant with a *p*-value of > 0.1 to avoid missing any important interactions.

IBM SPSS version 23.0 (IBM Corp, Somers, NY, USA) and STATA 13.1 Data Analysis and Statistical Software (StataCorp LP, Texas, USA) were used for statistical analysis.

## Results

### Participant characteristics

Mean (SD) follow-up was 366 (8) days. Table 1 and Additional file 1 provide the participant characteristics according to ulceration status at follow-up. Prevalence data for foot complications have been reported elsewhere [14]. Frequency data for primary and secondary outcomes are shown in Table 2 and Additional file 2. Foot examination, foot-health care behaviors and podiatry attendance according to ulceration status at follow-up are presented in Additional file 3.

**Table 2:** Primary and secondary outcomes according to foot ulceration status at follow-up

	Total (*N* = 450)	Foot ulceration		
		Yes (*n* = 81)	No (*n* = 369)	*P*-value*
Foot ulceration, n (%)^a^	81 (18)	81 (100)	N/A	N/A
Total number of new foot ulcers^b^	211	211		
New, total no. (%)	200 (95)	200 (95)		
Reoccurring, total no. (%)	11 (5)	11 (5)		
Time to onset of first foot ulcer, mean (SD), days	164 (127)	164 (127)		
New lower extremity amputation, n (%)	12 (3)	12 (15)	0 (0)	
Minor, n (%)	12 (3)	12 (15)	0 (0)	
Major, n (%)	2 (0.4)	2 (3)	0 (0)	
Total number of amputations^b^	20	20	N/A	
Minor, total no. (%)^c^	18 (90)	18 (90)		
Major, total no. (%)^d^	2 (10)	2 (10)		
Reason for amputation				
Infected foot ulcer, total no. (%)	8 (40)	8 (40)		
PAD/gangrene, total no. (%)	9 (45)	9 (45)		
Osteomyelitis, total no. (%)	3 (15)	3 (15)		
Time to first amputation, mean (SD), days	202 (104)	202 (104)		
Episodes of lower limb/foot infection, n (%)	96 (21)	53 (65)	43 (12)	< 0.001*
Total number of infections^b^	182	130	52	< 0.001*
Episodes of osteomyelitis, n (%)	24 (5)	23 (28)	1 (0.3)	< 0.001*
Foot-related hospitalizations, n (%)	42 (9)	35 (43)	7 (2)	< 0.001*
Total number of hospitalizations^b^	74	66	8	0.08
Reason for hospital admission				
Infected foot ulcer, n (%)	17 (4)	16 (20)	1 (0.3)	0.10
Lower extremity amputation, n (%)	5 (1)	4 (5)	1 (0.3)	> 0.99
Lower extremity revascularization procedure, n (%)	8 (2)	4 (5)	4 (1)	0.09
PAD/gangrene, n (%)	9 (2)	8 (10)	1 (0.3)	0.69
Cellulitis, n (%)	7 (2)	4 (5)	3 (0.8)	0.31
Osteomyelitis, n (%)	4 (0.9)	4 (5)	0 (0)	0.65
Other, n (%)	10 (2)	8 (10)	2 (0.5)	> 0.99
Length of stay, mean (SD), days	25 (23)	28 (23)	10 (9)	0.002*
Lower extremity revascularization procedure, n (%)	24 (5)	20 (25)	4 (1)	< 0.001*
Total number of lower extremity revascularization procedures^b^	42	37	5	0.18
Angioplasty, total no. (%)	34 (81)	30 (81)	4 (80)	Omitted
Bypass, total no. (%)	2 (5)	2 (5)	0 (0)	Omitted
Stent, total no. (%)	6 (14)	5 (14)	1 (20)	Omitted
New podiatry attendance, n (%)	38 (8)	13 (16)	25 (7)	< 0.001*
Kidney transplantation, n (%)	30 (7)	5 (6)	25 (7)	> 0.99
Time to transplant, mean (SD), days	195 (115)	191 (89)	196 (121)	0.91
All-cause mortality, n (%)	52 (12)	14 (17)	38 (10)	0.11
Foot-related death, n (%)^e^	6 (12)	5 (36)	1 (3)	0.005*
Sepsis due to infected foot ulcer, n (%)^e^	5 (10)	5 (36)	0 (0)	0.001*
Complications of PAD, n (%)^e^	1 (2)	0 (0)	1 (3)	> 0.99
Other causes of death, n (%)^e^	46 (88)	9 (64)	37 (97)	0.005*
Myocardial infarction, n (%)^e^	10 (19)	3 (21)	7 (18)	> 0.99
Withdrawal from dialysis, n (%)^e^	8 (15)	2 (14)	6 (16)	> 0.99
Pneumonia, n (%)^e^	8 (15)	0 (0)	8 (21)	0.15
Sepsis (not foot-related), n (%)^e^	5 (10)	0 (0)	5 (13)	0.37
Intestinal necrosis, n (%)^e^	3 (6)	1 (7)	2 (5)	> 0.99
ESRD, n (%)^e^	3 (6)	1 (7)	2 (5)	> 0.99
Other, n (%)^e^	9 (17)	2 (14)	7 (18)	> 0.99
Time to death, mean (SD), days	193 (115)	192 (112)	194 (118)	0.96

Data are n (%), unless otherwise specified. Percentages may not add up to 100%, as they are rounded to the nearest percent

The complete dataset of primary and secondary outcomes can be found in Additional file 2

*SD* Standard deviation, *PAD* Peripheral arterial disease, *ESRD* End-stage renal disease

*Significant difference between ‘foot ulceration’ and ‘no foot ulceration’ groups, *p* < 0.05

^a^Includes new and reoccurring foot ulcers. Reoccurring ulcers were in 9 participants (2.0%)

^b^Total number

^c^Minor amputations included: 10 single toe, 4 multiple toes, 1 partial toe, 1 single toe and metatarsal, and 2 transmetatarsal amputations

^d^Major amputations included: 2 below knee amputations

^e^Percentage calculated from all-cause mortality data

### Primary outcome

New foot ulceration was identified in 81 (18.0%) participants (Fig. 1). Of these, new foot ulceration occurred in 67/398 (16.8%) participants who were alive at the 12-month follow-up and 14/52 (26.9%) participants who died during the study period (new foot ulceration in 5/6 with foot-related death and 9/46 with other causes of death). Mean time to onset of first ulcer was 164 (SD, 127) days. Annual incidence of ulceration was 122 per 1000 person-years with 211 new ulcers in total (200 new and 11 reoccurring), the majority 128/211 (60.7%) were located on the toes (Table 3).

**Table 3:** Characteristics of foot ulcers and amputations

	Foot ulceration				Amputation		
	Total (*n* = 81)	Left (*n* = 48)	Right (*n* = 55)		Total (*n* = 12)	Left (*n* = 8)	Right (*n* = 5)
Foot ulcers, total no. (%)	211 (100)	103 (49)	108 (51)	Amputations, total no. (%)	20 (100)	11 (55)	9 (45)
New, total no. (%)	200 (95)	97 (95)	103 (95)	Minor, total no. (%)^a^	18 (90)	11 (100)	7 (78)
Reoccurring, total no. (%)	11 (5)	6 (6)	5 (2)	Major, total no. (%)^a^	2 (10)	0 (0)	2 (22)
Foot ulcers per participant				Amputations per participant			
Mean (SD), range	2.6 (2.0), 1 to 11	2.0 (1.2), 1 to 5	1.9 (1.2), 1 to 6	Mean (SD), range	1.7 (0.8), 1 to 3	0.9 (0.9), 0 to 3	0.8 (1.1), 0 to 3
Median (IQR)	2 (1 to 4)	2 (1 to 3)	1 (1 to 2)	Median (IQR)	1.5 (1 to 2)	1.0 (0 to 1)	0 (0 to 1.3)
Location^b^				Location			
Toes (dorsal, lateral or medial), total no. (%)	124 (59)	65 (63)	59 (55)	Single toe, total no. (%)	10 (50)	7 (64)	3 (33)
Plantar toes, forefoot, midfoot, total no. (%)	41 (19)	18 (18)	23 (21)	Multiple toes, total no. (%)	4 (20)	2 (18)	2 (22)
Dorsal foot, total no. (%)	14 (7)	7 (7)	7 (7)	Partial toe, total no. (%)	1 (5)	1 (9)	0 (0)
Heel, total no. (%)	32 (15)	13 (13)	19 (18)	Metatarsal + single toe, total no. (%)	1 (5)	0 (0)	1 (11)
				Transmetatarsal, total no. (%)	2 (10)	1 (9)	1 (11)
				Below knee amputation, total no. (%)	2 (10)	0 (0)	2 (22)
Type				Reason for amputation			
Neuropathic, total no. (%)	23 (11)	16 (16)	7 (7)	Infected foot ulcer, total no. (%)	8 (40)	4 (36)	4 (44)
Neuro-ischemic, total no. (%)	129 (61)	61 (59)	68 (63)	PAD/gangrene, total no. (%)	9 (45)	5 (46)	4 (44)
Ischemic, total no. (%)	10 (5)	5 (5)	5 (5)	Osteomyelitis, total no. (%)	3 (15)	2 (18)	1 (11)
Pressure injury, total no. (%)	32 (15)	13 (13)	19 (18)				
Other/unknown, total no. (%)	17 (8)	8 (8)	9 (8)				
Time to first foot ulcer, mean (SD), days	164 (127)	178 (124)	182 (125)	Time to first amputation, mean (SD), days	202 (104)	253 (90)	130 (79)

Data are total number of foot ulcers or amputations (%), unless otherwise specified. Percentages may not add up to 100%, as they are rounded to the nearest percent

*SD* Standard deviation, *IQR* Interquartile range, *PAD* Peripheral arterial disease

^a^Amputations were classified as ‘minor’ if below the ankle, or ‘major’ if above the ankle

^b^Majority of foot ulcers 128/211 (60.7%) were located on the toes (dorsal, lateral, medial or plantar aspects)

### Secondary outcomes

Among the 450 participants, 12 (2.7%) had at least one new amputation, with 20 amputations in total (18 minor and 2 major). The majority occurred due to peripheral arterial disease and/or gangrene (45.0%), infected foot ulcers (40.0%), and osteomyelitis (15.0%) (Tables 2 and 3).

Over 20% of participants (*n* = 96) had ≥1 foot or leg infection (182 episodes in total), including cellulitis (10.9%) and local wound infection (8.2%). Osteomyelitis occurred in 24 (5.3%), and 42 (9.3%) were admitted to hospital at least once for foot-related issues (74 admissions in total). The mean length of stay was 25 (SD, 23) days, with foot ulcer infection (28.4%) the most common reason for hospitalization. Revascularization procedures of the lower extremity (42 procedures in total) were performed on 24 (5.3%), the majority being angioplasties (81.0%) (Table 2).

Fifty-two (11.6%) died, the most common causes being myocardial infarction (23.1%), withdrawal from dialysis (15.4%), and pneumonia (15.4%). Specifically, six participants died from foot-related consequences: five from systemic sepsis secondary to an infected foot ulcer, and one from complications of peripheral arterial disease (Table 2).

### Risk factors for foot ulceration

Additional file 4 presents the risk factors that were significant in the univariate Cox proportional hazard model for foot ulceration. Risk factors with greatest hazard were previous lower extremity amputation (HR 6.52, 95% CI 2.83 to 14.99) and peripheral neuropathy (HR 4.14, 95% CI 1.99 to 8.61) (Additional file 5 – Kaplan-Meier survival estimates). Diabetes mellitus was not found to be a significant risk factor (HR 1.24, 95% CI 0.66 to 2.33), however stratification by diabetes status indicated modification of effects of other risk factors.

A subset of these risk factors were selected for inclusion in the multivariate Cox proportional hazard model based on their contribution to the maximum log partial likelihood and the statistical significance of the risk factor at *p* < 0.05 and exclusion at *p* > 0.1. In a multivariate analysis, peripheral neuropathy (HR 3.02, 95% CI 1.48 to 6.15, *p* = 0.002), previous foot ulceration (HR 2.86, 95% CI 1.53 to 5.34, *p* = 0.001) and cerebrovascular disease (HR 1.82, 95% CI 0.98 to 3.36, *p* = 0.057) remained as significant risk factors (Table 4).

**Table 4:** Multivariate Cox proportional hazard model of risk factors for foot ulceration

Risk factor	HR (95% CI)	*P*-value*
Peripheral neuropathy	3.02 (1.48 to 6.15)	0.002*
Previous foot ulceration	2.86 (1.53 to 5.34)	0.001*
Cerebrovascular disease	1.82 (0.98 to 3.36)	0.06

Analysis only includes participants without an ulcer at baseline

*HR* Hazard ratio, *CI* Confidence interval

*Significant risk factor, *p* < 0.05

Results of multinomial regression analyses are shown in Table 5. In those *without* a history of ulceration, nail pathology (RR 3.85, 95% CI 1.08 to 13.75) and neuropathy (RR 2.66, 95% CI 1.04 to 6.82) were significant risk factors. In those *with* history of ulceration, neuropathy (RR 11.23, 95% CI 3.16 to 39.87), peripheral arterial disease (RR 7.15, 95% CI 2.24 to 22.82), and cerebrovascular disease (RR 2.08, 95% CI 1.04 to 4.16) were significant.

**Table 5:** Multinomial regression analysis of risk factors for foot ulceration

Primary outcome	Category	N	Risk factor	RR (95% CI)	*P*-value*†
No development of foot ulceration	(i) No previous or baseline ulceration, and did not develop ulceration during study period	369	Reference Category		
Development of foot ulceration^a^	(ii) No previous or baseline ulceration, but developed ulceration during study period	27	Diabetes mellitus	0.68 (0.28 to 1.63)	0.39
			Neuropathy	2.66 (1.04 to 6.82)	0.04*
			Peripheral arterial disease	0.58 (0.24 to 1.41)	0.23
			Cerebrovascular disease	1.37 (0.54 to 3.50)	0.51
			Nail pathology	3.85 (1.08 to 13.75)	0.04*
	(iii) Previous and/or baseline ulceration, and developed ulceration during study period	54	Diabetes mellitus	1.84 (0.75 to 4.48)	0.18
			Neuropathy	11.23 (3.16 to 39.87)	< 0.001†
			Peripheral arterial disease	7.15 (2.24 to 22.82)	0.001†
			Cerebrovascular disease	2.08 (1.04 to 4.16)	0.04*
			Nail pathology	1.02 (0.43 to 2.45)	0.95

Adjusted for age, sex, living alone, podiatry attendance and diabetes

*RR* Relative risk, *CI* Confidence interval

*Significant risk factor, *p* < 0.05

†Significant risk factor, *p* < 0.01

^a^Includes new and reoccuring foot ulcers

## Discussion

Being alive, ulcer free and with limbs intact are important patient-related outcomes [15]. This study identified 211 foot ulcers in 450 stable dialysis patients over 12 months. Twelve participants required amputation and 6 participants died from foot-related complications. There were 74 hospital admissions (average of 25 days/admission) and 24 cases of osteomyelitis. Overall, in our sample of 450 participants, nearly a third (26.4%) had either died, developed an ulcer or had a lower limb amputation at 12 months.

Peripheral neuropathy and previous foot ulceration were found to be major risk factors for the development of foot ulceration, which is consistent with other studies [14, 16–19] and our previous meta-analysis [11]. These findings add to existing retrospective and cross-sectional studies by demonstrating a temporal association between these risk factors and foot ulceration. It is notable that peripheral neuropathy increased the risk of ulceration 3-fold. The sensory, motor and autonomic components of diabetic and/or uremic polyneuropathy often result in unnoticed injuries, muscle atrophy with associated foot deformity, and drying/fissuring of the skin [20, 21]. It is also notable that those with a history of ulceration were nearly 3-fold more likely to develop foot ulceration, as these patients often have the same risk factors that contributed to the original ulcer.

Although cerebrovascular disease had borderline significance (*p* = 0.06) in the multivariate analysis (confounded by neuropathy and previous ulceration), it was found to be a risk factor for ulceration in the multinomial analysis, specifically for those *with* a history of ulceration. A previous study [22] supports our finding that cerebrovascular disease may be an important risk factor for ulceration in dialysis patients (OR 2.78, 95% CI 1.02 to 7.62). This may be explained by a high prevalence of underlying atherosclerosis and microangiopathy with an associated reduction in cognition or functional status in dialysis patients with cerebrovascular disease [22]. This in turn may affect adherence to foot care or attendance to podiatry services. In addition, cerebrovascular disease may increase risk of falls and subsequent foot trauma or injury [20], so its relevance should not be discounted.

There were three additional important findings from our study. First, multinomial regression found that risk factors differ between those with and without prior ulceration. In those *without* a history of ulceration, nail pathology (RR 3.85) and neuropathy (RR 2.66) were risk factors. Whereas, in those *with* a history of ulceration, neuropathy (RR 11.23), peripheral arterial disease (RR 7.15), and cerebrovascular disease (RR 2.08) were dominant risk factors. However, these findings should be interpreted with caution as only 27 cases without past or baseline ulceration developed a foot ulcer during the study period.

Second, diabetes was not found to be a significant risk factor in our multivariate Cox proportional hazard or multinomial regression analysis, which differs from the results of our previous meta-analysis [11], where diabetes increased the risk of ulceration by 3.76-fold. This discrepancy may be explained by some of the limitations of the systematic review including: small sample sizes, unavailability of raw data, unexplained between-study heterogeneity, and a greater risk of confounding from measured and unmeasured factors (unadjusted risk factor data were sourced from non-randomized studies) [11]. The finding that diabetes was not a significant risk factor for ulceration in the current study is, however, consistent with our previous work [14] (OR 2.13, 95% CI 0.71 to 6.36), and suggests that much of the effect of diabetes on the risk of foot ulceration is mediated by coexisting neuropathy and/or peripheral arterial disease. In addition, diabetes confounded and was an effect modifier for both neuropathy and peripheral arterial disease. These results are also similar to our previous cross-sectional study [14], where diabetes was found to be a strong effect modifier (although particularly in men) for inter-related risk factors. Therefore, its existence in the dialysis population remains relevant and should not be discounted when establishing risk. Importantly, the study found a high rate of peripheral sensory neuropathy in participants with (66%) and without diabetes (35%), and that neuropathy was a strong risk factor for ulceration. As previously reported [14], 70 participants (15.6%) had peripheral neuropathy documented in their medical records prior to the baseline assessment. Remarkably, half the cohort (50.7%) were found to have peripheral sensory neuropathy on examination. This finding highlights that uremic neuropathy may be underdiagnosed, and provides further impetus for regular foot examination, where peripheral neuropathy is assessed, in the dialysis population [14].

Third, our study highlights a high annual incidence of foot ulceration in the dialysis population (122 events per 1000 person-years). Significantly, this is greater than two previous retrospective studies [2, 16], which may reflect issues of data recall and missing data in those studies. In addition, we found high rates of new lower extremity amputations, episodes of infection, revascularization procedures, and foot-related hospital admissions, which are generally comparable to previous studies [2, 3]. A Cox proportional hazard analysis for amputation (our secondary outcome) was not performed due to insufficient numbers (*n* = 12), most likely due to the limited follow-up time.

There are several potential limitations of this study. First, we did not exclude participants with a history of ulceration, as we wanted to establish the prevalence of previous/current ulceration, as well as the incidence of new ulceration. This was addressed by excluding those with a baseline ulcer in the Cox proportional hazards analysis, and the multinomial analysis compared participants according to ulceration status (previous/current) at baseline. Second, despite our best efforts to recruit a representative sample of dialysis patients, our cohort was largely from satellite (hospital-based) dialysis units, with the majority undertaking hemodialysis. Third, recall bias may have been present (e.g. participants self-reported new foot ulcers), however medical records were reviewed and health care providers were contacted if clarification was needed, so this was unlikely. Fourth, it was not possible to distinguish between different subtypes of peripheral neuropathy or other neuropathic conditions (e.g. diabetic amyotrophy) as non-invasive neurological assessments were used. This study focused on identifying the presence/extent of peripheral ‘sensory’ neuropathy, which from a clinical perspective, is considered the most important issue in establishing foot ulcer risk. Fifth, it is uncertain whether the presence of peripheral arterial disease may have been overestimated, particularly for toe- and ankle-brachial pressure indices, as previous small studies have indicated that cutaneous microcirculation may be affected during dialysis [23, 24]. To address this, foot assessments were conducted on participants prior to dialysis or on a non-dialysis day [13], however arterial assessments were mostly performed during dialysis treatment. In addition, footwear assessment was performed on shoes worn by participants at their baseline appointment, which may not have been representative. Finally, it was not possible to control for all potential confounding interventions that participants may have received from other sources.

There are several strengths of this study. It was adequately powered, and the large sample size, multi-center recruitment and inclusion of a full range of risk factors allow the findings to be generalized to clinical practice. No participants were lost to follow-up, so a complete data set was analyzed. Finally, the prospective study design has established, for the first time, a temporal association between screened risk factors and an increase in foot ulceration in dialysis patients.

Our study highlights a clear need for foot care provision to dialysis patients, either with or without the presence of diabetes. Given that those with peripheral neuropathy and/or previous ulceration have an approximately 3-fold risk of new ulceration, dialysis patients may benefit from strategies to prevent foot complications, such as regular foot screening and early intervention. Further research is needed to evaluate the effectiveness of these strategies.

## Conclusions

This study is the first to identify longitudinal risk estimates for foot ulceration in a large dialysis cohort. Risk factors differ between those with and without a history of ulceration, however adults on dialysis with peripheral neuropathy and previous foot ulceration are at highest risk of developing foot ulcers. Diabetes is not itself a significant risk factor as other comorbidities, such as neuropathy and peripheral arterial disease, have stronger associations with ulceration. These findings will help reduce the incidence of foot ulceration and its associated complications.

## Supplementary information

**Additional file 1.** Complete dataset of participant characteristics according to foot ulceration status at follow-up. Table showing the complete dataset of participant characteristics according to foot ulceration status at follow-up.

**Additional file 2.** Complete dataset of primary and secondary outcomes according to foot ulceration status at follow-up. Table showing the complete dataset of primary and secondary outcomes according to foot ulceration status at follow-up.

**Additional file 3.** Foot examination, foot-health care behaviors and podiatry attendance according to foot ulceration status at follow-up. Table showing data relating to foot examination, foot-health care behaviors and podiatry attendance according to foot ulceration status at the 12-month follow-up.

**Additional file 4.** Univariate Cox proportional hazard model of risk factors for foot ulceration stratified by diabetes status. Table showing the results of a Univariate Cox proportional hazard model of risk factors for foot ulceration stratified by diabetes status.

**Additional file 5.** Kaplan-Meier survival estimates by (a) previous lower extremity amputation and (b) peripheral neuropathy. Figure showing Kaplan-Meier survival estimates by (a) previous lower extremity amputation and (b) peripheral neuropathy.

## Data Availability

The datasets used and/or analyzed during the current study are available from the corresponding author on reasonable request.

## Abbreviations

APD: Automated peritoneal dialysis
BMI: Body mass index
CAPD: Continuous ambulatory peritoneal dialysis
CI: Confidence interval
CRP: C-reactive protein
ESRD: End-stage renal disease
HbA1c: Glycated hemoglobin
HR: Hazard ratio
IQR: Interquartile range
MCS: Mental component score
MD: Mean difference
OR: Odds ratio
PAD: Peripheral arterial disease
PCS: Physical component score
PTH: Parathyroid hormone
RR: Relative risk
SD: Standard deviation
SF-36v2: Short-Form 36 version 2.0

## References

[1] Al-Thani H, El-Menyar A, Koshy V, Hussein A, Sharaf A, Asim M, Sadek A (2014). Implications of foot ulceration in hemodialysis patients: a 5-year observational study. J Diabetes Res.

[2] Lavery LA, Lavery DC, Hunt NA, La Fontaine J, Ndip A, Boulton AJ (2013). Amputations and foot-related hospitalisations disproportionately affect dialysis patients. Int Wound J.

[3] Lavery LA, Hunt NA, Ndip A, Lavery DC, Van Houtum W, Boulton AJM (2010). Impact of chronic kidney disease on survival after amputation in individuals with diabetes. Diabetes Care.

[4] Jaar BG, Astor BC, Berns JS, Powe NR (2004). Predictors of amputation and survival following lower extremity revascularization in hemodialysis patients. Kidney Int.

[5] Boulton AJM, Vileikyte L, Ragnarson-Tennvall G, Apelqvist J (2005). The global burden of diabetic foot disease. Lancet.

[6] Orimoto Y, Ohta T, Ishibashi H, Sugimoto I, Iwata H, Yamada T, Tadakoshi M, Hida N (2013). The prognosis of patients on hemodialysis with foot lesions. J Vasc Surg.

[7] Larsson J, Stenström A, Apelqvist J, Agardh CD (1995). Decreasing incidence of major amputation in diabetic patients: a consequence of a multidisciplinary foot care team approach?. Diabet Med.

[8] Bus SA, van Netten JJ (2016). A shift in priority in diabetic foot care and research: 75% of foot ulcers are preventable. Diabetes Metab Res Rev.

[9] Abbott CA, Johnson KE, Ryder CH, Torkington R, Van Ross ERE, Whalley AM, Widdows P, Williamson S, Boulton AJM, Carrington AL (2002). The north-west diabetes foot care study: incidence of, and risk factors for, new diabetic foot ulceration in a community-based patient cohort. Diabet Med.

[10] Boyko EJ, Ahroni JH, Cohen V, Nelson KM, Heagerty PJ (2006). Prediction of diabetic foot ulcer occurrence using commonly available clinical information: the Seattle diabetic foot study. Diabetes Care.

[11] Kaminski MR, Raspovic A, McMahon LP, Strippoli GFM, Palmer SC, Ruospo M, Dallimore S, Landorf KB (2015). Risk factors for foot ulceration and lower extremity amputation in adults with end-stage renal disease on dialysis: a systematic review and meta-analysis. Nephrol Dial Transplant.

[12] O’Hare AM, Feinglass J, Reiber GE, Rodriguez RA, Daley J, Khuri S, Henderson WG, Johansen KL (2004). Postoperative mortality after nontraumatic lower extremity amputation in patients with renal insufficiency. J Am Soc Nephrol.

[13] Kaminski MR, Raspovic A, McMahon LP, Erbas B, Landorf KB (2015). Risk factors for foot ulceration in adults with end-stage renal disease on dialysis: study protocol for a prospective observational cohort study. J Foot Ankle Res.

[14] Kaminski MR, Raspovic A, McMahon LP, Lambert KA, Erbas B, Mount PF, Kerr PG, Landorf KB (2017). Factors associated with foot ulceration and amputation in adults on dialysis: a cross-sectional observational study. BMC Nephrol.

[15] Jeffcoate WJ, Chipchase SY, Ince P, Game FL (2006). Assessing the outcome of the management of diabetic foot ulcers using ulcer-related and person-related measures. Diabetes Care.

[16] Otte J, van Netten JJ, Woittiez AJ (2015). The association of chronic kidney disease and dialysis treatment with foot ulceration and major amputation. J Vasc Surg.

[17] Ndip A, Rutter MK, Vileikyte L, Vardhan A, Asari A, Jameel M, Tahir HA, Lavery LA, Boulton AJ (2010). Dialysis treatment is an independent risk factor for foot ulceration in patients with diabetes and stage 4 or 5 chronic kidney disease. Diabetes Care.

[18] Ndip A, Lavery LA, Lafontaine J, Rutter MK, Vardhan A, Vileikyte L, Boulton AJ (2010). High levels of foot ulceration and amputation risk in a multiracial cohort of diabetic patients on dialysis therapy. Diabetes Care.

[19] Pliakogiannis T, Bailey S, Cherukuri S, Taskapan H, Ahmad M, Oliver T, Bargman JM, Oreopoulos DG (2008). Vascular complications of the lower extremities in diabetic patients on peritoneal dialysis. Clin Nephrol.

[20] Lewis S, Raj D, Guzman NJ (2012). Renal failure: implications of chronic kidney disease in the management of the diabetic foot. Semin Vasc Surg.

[21] Ndip A, Lavery LA, Boulton AJM (2010). Diabetic foot disease in people with advanced nephropathy and those on renal dialysis. Curr Diab Rep.

[22] Yasuhara H, Naka S, Yanagie H, Nagawa H (2002). Influence of diabetes on persistent nonhealing ischemic foot ulcer in end-stage renal disease. World J Surg.

[23] Weiss T, Windthorst C, Weiss C, Kreuzer J, Bommer J, Kübler W (1998). Acute effects of haemodialysis on cutaneous microcirculation in patients with peripheral arterial occlusive disease. Nephrol Dial Transplant.

[24] Hinchliffe RJ, Kirk B, Bhattacharjee D, Roe S, Jeffcoate W, Game F (2006). The effect of haemodialysis on transcutaneous oxygen tension in patients with diabetes - a pilot study. Nephrol Dial Transplant.

