# Supplementary material for: Risk factors for foot ulceration in adults with end-stage renal disease on dialysis: a prospective observational cohort study
Source: BMC Nephrol. 2019 Nov 21;20:423. doi: 10.1186/s12882-019-1594-5 (PMC6868750; doi:10.1186/s12882-019-1594-5)
Supplement: Supplementary file 2 — Additional file 2. Complete dataset of primary and secondary outcomes according to foot ulceration status at follow-up. Table showing the complete dataset of primary and secondary outcomes according to foot ulceration status at follow-up. [file 12882_2019_1594_MOESM2_ESM.pdf]

RISK FACTORS FOR FOOT ULCERATION IN ADULTS WITH END-STAGE RENAL DISEASE ON DIALYSIS: A PROSPECTIVE OBSERVATIONAL COHORT STUDY

Michelle R Kaminski, Katrina A Lambert, Anita Raspovic, Lawrence P McMahon, Bircan Erbas, Peter F Mount, Peter G Kerr, Karl B Landorf

---

Additional File 1 Complete dataset of participant characteristics according to foot ulceration status at follow-up

		Foot ulceration		
	**Total (N = 450)**	**Yes (n = 81)**	**No (n = 369)**	**P-value***
Time to follow-up, mean (SD), days	366 (8)	366 (7)	366 (9)	0.48
Age, mean (SD), years	68 (13)	69 (10)	67 (14)	0.33
Male sex, n (%)	291 (65)	56 (69)	235 (64)	0.42
BMI, mean (SD), kg/m²	28.2 (6.6)	29.9 (7.0)	27.8 (6.4)	0.01*
Current smoking, n (%)	54 (12)	9 (11)	45 (12)	0.93
Living alone, n (%)	75 (17)	11 (14)	64 (17)	0.51
Ethnicity				
Indigenous Australian, n (%)	4 (0.9)	1 (1)	3 (0.8)	>0.99
English, n (%)	85 (19)	14 (17)	71 (19)	0.80
European, n (%)	194 (43)	34 (42)	160 (43)	0.92
American, n (%)	4 (0.9)	1 (1)	3 (0.8)	>0.99
African, n (%)	14 (4)	3 (4)	11 (3)	>0.99
Asian, n (%)	67 (15)	8 (10)	59 (16)	0.22

Pacific Islander, n (%)	29 (6)	8 (10)	21 (6)	0.26
Other, n (%)	53 (12)	12 (15)	41 (11)	0.46
Cause of ESRD				
Diabetes mellitus, n (%)	180 (40)	53 (65)	127 (34)	<0.001*
Hypertension, n (%)	28 (6)	4 (5)	24 (7)	0.78
Glomerulonephritis, n (%)	97 (22)	11 (14)	86 (23)	0.08
Polycystic kidney disease, n (%)	22 (5)	4 (5)	18 (5)	>0.99
Reflux, n (%)	19 (4)	1 (1)	18 (5)	0.24
Renovascular disease, n (%)	10 (2)	3 (4)	7 (2)	0.56
Vasculitis, n (%)	9 (2)	1 (1)	8 (2)	0.92
Unknown, n (%)	15 (3)	0 (0)	15 (4)	0.13
Other, n (%)	70 (16)	4 (5)	66 (18)	0.006*
Dialysis treatment				
Hemodialysis, n (%)	423 (94)	79 (98)	344 (93)	0.85
Peritoneal dialysis				
CAPD, n (%)	9 (2)	1 (1)	8 (2)	0.92
APD, n (%)	18 (4)	1 (1)	17 (5)	>0.99
Dialysis duration, median (IQR), months	36.9 (16.6 to 70.1)	41.3 (19.7 to 82.1)	36.4 (14.7 to 67.4)	0.28
Diabetes, n (%)	226 (50)	58 (72)	168 (46)	<0.001*
Type 1, n (%)	13 (6)	6 (7)	7 (2)	0.16
Type 2, n (%)	213 (94)	52 (64)	161 (44)	0.16
Diabetes duration, mean (SD), months	256.3 (152.6)	311.7 (157.2)	237.1 (146.6)	0.002*
Retinopathy, n (%)	132 (29)	41 (51)	91 (25)	<0.001*
Known peripheral neuropathy, n (%)	70 (16)	31 (38)	39 (11)	<0.001*
Known peripheral arterial disease, n (%)	79 (18)	36 (44)	43 (12)	<0.001*

Lower extremity revascularization procedure, n (%)	35 (8)	20 (25)	15 (4)	<0.001*
Hypertension, n (%)†	360 (80)	66 (82)	294 (80)	0.83
Dyslipidemia, n (%)	301 (67)	64 (79)	237 (64)	0.02*
Ischemic heart disease, n (%)	263 (58)	58 (72)	205 (56)	0.01*
Congestive cardiac failure, n (%)	122 (27)	29 (36)	93 (25)	0.07
Cerebrovascular disease, n (%)	104 (23)	31 (38)	73 (20)	0.001*
Osteoarthritis, n (%)	192 (43)	36 (44)	156 (42)	0.82
Inflammatory arthritis, n (%)	183 (41)	35 (43)	148 (40)	0.70
CRP, median (IQR), mg/L‡	7.33 (2.83 to 19.67)	10.33 (4.65 to 24.38)	6.67 (2.67 to 18.75)	0.03
Serum albumin, mean (SD), g/L	33.7 (3.9)	32.8 (4.9)	33.9 (3.7)	0.06
Total calcium, mean (SD), mmol/L	2.20 (0.14)	2.20 (0.15)	2.20 (0.13)	0.86
Serum phosphate, mean (SD), mmol/L	1.55 (0.38)	1.60 (0.44)	1.54 (0.37)	0.27
PTH, median (IQR), pmol/L	29.58 (18.04 to 45.84)	27.53 (21.17 to 45.97)	29.83 (16.93 to 45.65)	0.46
HbA1c, mean (SD), %‡	6.14 (1.31)	6.65 (1.35)	6.02 (1.28)	<0.001*
Hemoglobin, median (IQR), g/L	111.3 (102.9 to 117.7)	112.7 (102.8 to 120.2)	111.0 (102.8 to 117.3)	0.30
SF-36v2® PCS, mean (SD)	38.14 (10.70)	34.53 (9.96)	38.93 (10.71)	0.001*
SF-36v2® MCS, mean (SD)	48.55 (11.40)	46.12 (12.83)	49.08 (11.02)	0.06
Previous foot ulceration, n (%)	97 (22)	45 (56)	52 (14)	<0.001*
Baseline foot ulceration, n (%)	45 (10)	36 (44)	9 (2)	<0.001*
Baseline amputation, n (%)	46 (10)	30 (37)	16 (4)	<0.001*

Data are n (%), unless otherwise specified. Percentages may not add up to 100%, as they are rounded to the nearest percent.

SD, standard deviation; BMI, body mass index; ESRD, end-stage renal disease; CAPD, continuous ambulatory peritoneal dialysis; APD, automated peritoneal dialysis; IQR, interquartile range; CRP, C-reactive protein; PTH, parathyroid hormone; HbA1c, glycated hemoglobin; SF-36v2®, short-form-36 version 2.0; PCS, physical component score; MCS, mental component score.

SI conversion factor: To convert CRP to nanomoles per liter, multiply by 9.524. To convert PTH to nanograms per liter, multiply by 9.4. To convert HbA1c to proportion of total hemoglobin, multiply by 0.01.

*Significant difference between ‘foot ulceration’ and ‘no foot ulceration’ groups, $p < 0.05$.

†Requiring medication

‡Maximum missing data were for glycated hemoglobin (HbA1c) involving 39 participants overall (8.7%). Missing data were for glycated hemoglobin (n = 39) and C-reactive protein (n = 3).
