# Supplementary material for: Risk factors for foot ulceration in adults with end-stage renal disease on dialysis: a prospective observational cohort study
Source: BMC Nephrol. 2019 Nov 21;20:423. doi: 10.1186/s12882-019-1594-5 (PMC6868750; doi:10.1186/s12882-019-1594-5)
Supplement: Supplementary file 3 — Additional file 3. Foot examination, foot-health care behaviors and podiatry attendance according to foot ulceration status at follow-up. Table showing data relating to foot examination, foot-health care behaviors and podiatry attendance according to foot ulceration status at the 12-month follow-up. [file 12882_2019_1594_MOESM3_ESM.pdf]

RISK FACTORS FOR FOOT ULCERATION IN ADULTS WITH END-STAGE RENAL DISEASE ON DIALYSIS: A PROSPECTIVE OBSERVATIONAL COHORT STUDY

Michelle R Kaminski, Katrina A Lambert, Anita Raspovic, Lawrence P McMahon, Bircan Erbas, Peter F Mount, Peter G Kerr, Karl B Landorf

---

Additional File 2 Complete dataset of primary and secondary outcomes according to foot ulceration status at follow-up

		Foot ulceration		
	**Total (N = 450)**	**Yes (n = 81)**	**No (n = 369)**	***P*-value***
Foot ulceration, n (%)†	81 (18)	81 (100)	N/A	N/A
Total number of new foot ulcers‡	211	211		
New, total no. (%)	200 (95)	200 (95)		
Reoccurring, total no. (%)	11 (5)	11 (5)		
Time to onset of first foot ulcer, mean (SD), days	164 (127)	164 (127)		
New lower extremity amputation, n (%)	12 (3)	12 (15)	0 (0)	
Minor, n (%)	12 (3)	12 (15)	0 (0)	
Major, n (%)	2 (0.4)	2 (3)	0 (0)	
Total number of amputations‡	20	20	N/A	
Minor, total no. (%)§	18 (90)	18 (90)		
Major, total no. (%)‖	2 (10)	2 (10)		
Reason for amputation				
Infected foot ulcer, total no. (%)	8 (40)	8 (40)		
PAD/gangrene, total no. (%)	9 (45)	9 (45)		
Osteomyelitis, total no. (%)	3 (15)	3 (15)		
Time to first amputation, mean (SD), days	202 (104)	202 (104)		
Episodes of lower limb/foot infection, n (%)	96 (21)	53 (65)	43 (12)	<0.001*
Total number of infections‡	182	130	52	<0.001*
Type of infection				
Fungal infection, n (%)	23 (5)	4 (5)	19 (5)	<0.001*
Local wound infection, n (%)	37 (8)	35 (43)	2 (0.5)	<0.001*
Cellulitis, n (%)	49 (11)	29 (36)	20 (5)	0.55
Calciphylaxis and/or gangrene, n (%)	13 (3)	11 (14)	2 (0.5)	0.05*
Osteomyelitis, n (%)	24 (5)	23 (28)	1 (0.3)	<0.001*

Foot-related sepsis, n (%)	4 (0.9)	4 (5)	0 (0)	0.19
Other, n (%)	4 (0.9)	2 (3)	2 (0.5)	>0.99
Episodes of osteomyelitis, n (%)	24 (5)	23 (28)	1 (0.3)	<0.001*
Foot-related hospitalizations, n (%)	42 (9)	35 (43)	7 (2)	<0.001*
Total number of hospitalizations‡	74	66	8	0.08
Reason for hospital admission				
Infected foot ulcer, n (%)	17 (4)	16 (20)	1 (0.3)	0.10
Lower extremity amputation, n (%)	5 (1)	4 (5)	1 (0.3)	>0.99
Lower extremity revascularization procedure, n (%)	8 (2)	4 (5)	4 (1)	0.09
PAD/gangrene, n (%)	9 (2)	8 (10)	1 (0.3)	0.69
Cellulitis, n (%)	7 (2)	4 (5)	3 (0.8)	0.31
Osteomyelitis, n (%)	4 (0.9)	4 (5)	0 (0)	0.65
Other, n (%)	10 (2)	8 (10)	2 (0.5)	>0.99
Foot-related treatment/procedures				
Prescription of antibiotics, n (%)	35 (8)	31 (38)	4 (1)	0.14
Wound care/management, n (%)	33 (7)	31 (38)	2 (0.5)	0.002*
Lower extremity revascularization procedure, n (%)	24 (5)	20 (25)	4 (1)	>0.99
Surgical debridement, n (%)	10 (2)	10 (12)	0 (0)	0.26
Lower extremity amputation, n (%)	12 (3)	12 (15)	0 (0)	0.17
Issued an offloading device, n (%)	20 (4)	19 (24)	1 (0.3)	0.13
Podiatry treatment, n (%)	23 (5)	23 (28)	0 (0)	0.006*
Other, n (%)	26 (6)	23 (28)	3 (0.8)	0.48
Length of stay, mean (SD), days	25 (23)	28 (23)	10 (9)	0.002*
Lower extremity revascularization procedure, n (%)	24 (5)	20 (25)	4 (1)	<0.001*
Total number of lower extremity revascularization procedures‡	42	37	5	0.18
Angioplasty, total no. (%)	34 (81)	30 (81)	4 (80)	Omitted
Bypass, total no. (%)	2 (5)	2 (5)	0 (0)	Omitted
Stent, total no. (%)	6 (14)	5 (14)	1 (20)	Omitted
New podiatry attendance, n (%)	38 (8)	13 (16)	25 (7)	<0.001*
New podiatry interventions, n (%)	112 (25)	53 (65)	59 (16)	<0.001*
Nail/callus reduction, n (%)	36 (8)	14 (17)	22 (6)	0.25
Wound care/management, n (%)	38 (8)	35 (43)	3 (0.8)	<0.001*
Foot orthoses, n (%)	12 (3)	7 (9)	5 (1)	0.66
Issued an offloading device, n (%)	21 (5)	19 (23)	2 (0.5)	<0.001*
Padding/strapping, n (%)	23 (5)	18 (22)	5 (1)	0.003*
Footwear education, n (%)	33 (7)	16 (20)	17 (5)	>0.99

Foot care education, n (%)	34 (8)	17 (21)	17 (5)	0.96
Other, n (%)	35 (8)	13 (16)	22 (6)	0.17
Kidney transplantation, n (%)	30 (7)	5 (6)	25 (7)	>0.99
Time to transplant, mean (SD), days	195 (115)	191 (89)	196 (121)	0.91
All-cause mortality, n (%)	52 (12)	14 (17)	38 (10)	0.11
Foot-related death, n (%)¶	6 (12)	5 (36)	1 (3)	0.005*
Sepsis due to infected foot ulcer, n (%)¶	5 (10)	5 (36)	0 (0)	0.001*
Complications of PAD, n (%)¶	1 (2)	0 (0)	1 (3)	>0.99
Other causes of death, n (%)¶	46 (88)	9 (64)	37 (97)	0.005*
Myocardial infarction, n (%)¶	10 (19)	3 (21)	7 (18)	>0.99
Withdrawal from dialysis, n (%)¶	8 (15)	2 (14)	6 (16)	>0.99
Pneumonia, n (%)¶	8 (15)	0 (0)	8 (21)	0.15
Sepsis (not foot-related) , n (%)¶	5 (10)	0 (0)	5 (13)	0.37
Intestinal necrosis, n (%)¶	3 (6)	1 (7)	2 (5)	>0.99
ESRD, n (%)¶	3 (6)	1 (7)	2 (5)	>0.99
Other, n (%)¶	9 (17)	2 (14)	7 (18)	>0.99
Time to death, mean (SD), days	193 (115)	192 (112)	194 (118)	0.96

Data are n (%), unless otherwise specified. Percentages may not add up to 100%, as they are rounded to the nearest percent.

SD, standard deviation; PAD, peripheral arterial disease; ESRD, end-stage renal disease.

*Significant difference between 'foot ulceration' and 'no foot ulceration' groups, $p < 0.05$.

†Includes new and reoccurring foot ulcers. Reoccurring ulcers were in 9 participants (2.0%).

‡Total number.

§Minor amputations included: 10 single toe, 4 multiple toes, 1 partial toe, 1 single toe and metatarsal, and 2 transmetatarsal amputations.

‖Major amputations included: 2 below knee amputations.

¶Percentage calculated from all-cause mortality data.
