# Supplementary material for: Risk factors for foot ulceration in adults with end-stage renal disease on dialysis: a prospective observational cohort study
Source: BMC Nephrol. 2019 Nov 21;20:423. doi: 10.1186/s12882-019-1594-5 (PMC6868750; doi:10.1186/s12882-019-1594-5)
Supplement: Supplementary file 4 — Additional file 4. Univariate Cox proportional hazard model of risk factors for foot ulceration stratified by diabetes status. Table showing the results of a Univariate Cox proportional hazard model of risk factors for foot ulceration stratified by diabetes status. [file 12882_2019_1594_MOESM4_ESM.pdf]

RISK FACTORS FOR FOOT ULCERATION IN ADULTS WITH END-STAGE RENAL DISEASE ON DIALYSIS: A PROSPECTIVE OBSERVATIONAL COHORT STUDY

Michelle R Kaminski, Katrina A Lambert, Anita Raspovic, Lawrence P McMahon, Bircan Erbas, Peter F Mount, Peter G Kerr, Karl B Landorf

---

Additional File 3 Foot examination, foot-health care behaviors and podiatry attendance according to foot ulceration status at follow-up

		**Foot ulceration**		
	**Total (N = 450)**	**Yes (n = 81)**	**No (n = 369)**	***P*-value***
Peripheral neuropathy, n (%)	228 (50.7)	68 (84.0)	160 (43.4)	<0.001*
Peripheral arterial disease, n (%)	236 (52.4)	62 (76.5)	174 (47.2)	<0.001*
Arterial calcification, n (%)†	184 (40.9)	38 (46.9)	146 (39.6)	0.27
Foot deformity, n (%)	341 (75.8)	71 (87.7)	270 (73.2)	0.009*
Limited range of motion of first MTPJ, n (%)‡	421 (93.6)	76 (93.8)	345 (93.5)	0.07
Peak plantar pressure, median (IQR), kg/cm^2‡				
Total left foot	1.74 (1.50 to 2.06)	1.85 (1.48 to 2.29)	1.73 (1.51 to 2.01)	0.12
Total right foot	1.72 (1.50 to 2.09)	2.04 (1.51 to 2.26)	1.71 (1.50 to 2.03)	0.035*
Skin pathology, n (%)	395 (87.8)	73 (90.1)	322 (87.3)	0.60
Nail pathology, n (%)	319 (70.9)	68 (84.0)	251 (68.0)	0.006*
Inappropriate footwear, n (%)	297 (66.0)	52 (64.2)	245 (66.4)	0.80
Poor foot-health care, n (%)	136 (30.2)	25 (30.9)	111 (30.1)	>0.99
Podiatry attendance, last 12 months, n (%)	223 (49.6)	57 (70.4)	166 (45.0)	<0.001*

Data are n (%), unless otherwise specified. Percentages may not add up to 100%, as they are rounded to the nearest percent.

MTPJ, metatarsophalangeal joint; IQR, interquartile range.

*Significant difference between ‘foot ulceration’ and ‘no foot ulceration’ groups, $p < 0.05$.

†Lower extremity arterial calcification determined clinically using ankle-brachial pressure index >1.3 or non-compressible arteries (i.e. >240 mm Hg).

‡Maximum missing data were for left peak plantar pressure involving 56 participants overall (12.4%). Missing data were for limited range of motion of first MTPJ (left, n = 25; right, n = 15) and peak plantar pressures (left, n = 56; right, n = 55).
