# Supplementary material for: Risk factors for foot ulceration in adults with end-stage renal disease on dialysis: a prospective observational cohort study
Source: BMC Nephrol. 2019 Nov 21;20:423. doi: 10.1186/s12882-019-1594-5 (PMC6868750; doi:10.1186/s12882-019-1594-5)
Supplement: Supplementary file 5 — Additional file 5. Kaplan-Meier survival estimates by (a) previous lower extremity amputation and (b) peripheral neuropathy. Figure showing Kaplan-Meier survival estimates by (a) previous lower extremity amputation and (b) peripheral neuropathy. [file 12882_2019_1594_MOESM5_ESM.pdf]

RISK FACTORS FOR FOOT ULCERATION IN ADULTS WITH END-STAGE RENAL DISEASE ON DIALYSIS: A PROSPECTIVE OBSERVATIONAL COHORT STUDY

Michelle R Kaminski, Katrina A Lambert, Anita Raspovic, Lawrence P McMahon, Bircan Erbas, Peter F Mount, Peter G Kerr, Karl B Landorf

---

Additional File 4 Univariate Cox proportional hazard model of risk factors for foot ulceration stratified by diabetes status

	**Total (n = 405)**	***P*-value***	**No diabetes (n = 218)**	***P*-value***	**Diabetes (n = 187)**	***P*-value***
Diabetes mellitus	1.24 (0.66 to 2.33)	0.50				
BMI, kg/m²	1.04 (1.00 to 1.08)	0.04*	1.01 (0.94 to 1.10)	0.70	1.07 (1.02 to 1.14)	0.01*
Current smoking	0.78 (0.28 to 2.23)	0.65	0.68 (0.08 to 5.35)	0.71	0.86 (0.25 to 2.91)	0.81
Duration of dialysis, months	1.00 (0.99 to 1.01)	0.62	1.00 (0.99 to 1.01)	0.43	1.00 (0.99 to 1.01)	0.37
Retinopathy	1.64 (0.88 to 3.04)	0.12	Omitted	-	1.59 (0.71 to 3.58)	0.26
Peripheral neuropathy	4.14 (1.99 to 8.61)	0.001*	1.83 (0.66 to 5.06)	0.25	7.05 (2.08 to 23.91)	0.002*
Peripheral arterial disease	1.51 (0.79 to 2.85)	0.21	1.00 (0.38 to 2.63)	>0.99	1.72 (0.73 to 4.07)	0.22
Lower extremity arterial calcification	1.27 (0.70 to 2.32)	0.42	1.20 (0.47 to 3.09)	0.70	1.18 (0.54 to 2.55)	0.68
Hypertension†	0.79 (0.39 to 1.61)	0.53	0.64 (0.24 to 1.70)	0.37	0.68 (0.23 to 1.99)	0.48
Dyslipidemia	1.40 (0.70 to 2.84)	0.34	1.09 (0.43 to 2.78)	0.84	1.39 (0.41 to 4.70)	0.59
Ischemic heart disease	1.44 (0.74 to 2.78)	0.27	1.69 (0.65 to 4.44)	0.28	1.17 (0.47 to 2.87)	0.74
Congestive cardiac failure	1.30 (0.69 to 2.47)	0.42	1.26 (0.46 to 3.47)	0.65	1.43 (0.60 to 3.38)	0.42
Cerebrovascular disease	2.12 (1.14 to 3.92)	0.02*	2.06 (0.74 to 5.77)	0.17	2.36 (1.07 to 5.23)	0.03*
Osteoarthritis	1.27 (0.66 to 2.42)	0.47	1.48 (0.55 to 3.99)	0.44	1.19 (0.49 to 2.88)	0.69
Inflammatory arthritis	1.52 (0.84 to 2.75)	0.16	3.08 (1.09 to 8.72)	0.04*	0.97 (0.43 to 2.21)	0.95
CRP, mg/L	1.00 (0.99 to 1.01)	0.13	0.99 (0.97 to 1.01)	0.64	1.01 (1.00 to 1.02)	0.01*
Serum albumin, g/L	1.00 (0.93 to 1.08)	0.98	1.07 (0.95 to 1.21)	0.25	0.99 (0.90 to 1.09)	0.83
Total calcium, mmol/L	7.41 (0.80 to 68.41)	0.08	12.78 (0.54 to 30.17)	0.11	6.13 (0.29 to 131.6)	0.25
Mean phosphate, mmol/L	1.12 (0.52 to 2.45)	0.77	0.42 (0.09 to 2.02)	0.28	1.36 (0.53 to 3.46)	0.52
PTH, pmol/L	1.01 (1.00 to 1.01)	0.03*	1.01 (0.99 to 1.03)	0.20	1.00 (1.00 to 1.01)	0.27
HbA1c, %	1.08 (0.87 to 1.36)	0.47	0.91 (0.26 to 3.24)	0.89	0.99 (0.73 to 1.33)	0.93
Hemoglobin, g/L	1.03 (1.00 to 1.05)	0.03*	1.03 (0.99 to 1.08)	0.11	1.03 (0.99 to 1.06)	0.13

Previous foot ulceration	4.02 (2.15 to 7.52)	0.001*	2.42 (0.76 to 7.68)	0.13	4.37 (1.89 to 10.14)	0.001*
Lower extremity amputation	6.52 (2.83 to 14.99)	0.001*	Omitted	-	6.69 (2.44 to 18.34)	0.001*
Foot deformity	2.69 (1.05 to 6.91)	0.04*	1.48 (0.39 to 5.59)	0.56	4.65 (1.09 to 19.76)	0.04*
Peak plantar pressure, kg/cm^2						
Total left foot	1.10 (0.65 to 1.87)	0.72	0.56 (0.19 to 1.63)	0.29	1.76 (0.86 to 3.59)	0.12
Total right foot	1.15 (0.70 to 1.89)	0.58	0.54 (0.18 to 1.61)	0.27	2.27 (1.10 to 4.72)	0.03*
Skin pathology	1.02 (0.40 to 2.61)	0.96	1.17 (0.27 to 5.09)	0.84	0.77 (0.23 to 2.62)	0.68
Nail pathology	2.12 (0.92 to 4.88)	0.08	2.09 (0.57 to 7.63)	0.27	1.54 (0.52 to 4.56)	0.44
Inappropriate footwear	0.99 (0.52 to 1.90)	0.98	4.24 (0.96 to 18.82)	0.06	0.53 (0.24 to 1.18)	0.12
Poor foot-health care	1.95 (1.01 to 3.76)	0.05*	2.57 (0.96 to 6.88)	0.06	2.33 (0.90 to 5.99)	0.08

Values shown are relative risk of new foot ulcer expressed as HR (95% CI). Adjusted for age, male sex, living alone and podiatry attendance in the last 12 months. Analysis only includes participants without a baseline ulcer.

BMI, body mass index; CI, confidence interval; CRP, C-reactive protein; HR, hazard ratio; PTH, parathyroid hormone; HbA1c, glycated hemoglobin.

SI conversion factor: To convert CRP to nanomoles per liter, multiply by 9.524. To convert PTH to nanograms per liter, multiply by 9.4. To convert HbA1c to proportion of total hemoglobin, multiply by 0.01.

**P*-value < 0.05.

†Requiring medication.
